# Oral Delivery of Exenatide via Microspheres Prepared by Cross-Linking of Alginate and Hyaluronate

**DOI:** 10.1371/journal.pone.0086064

**Published:** 2014-01-21

**Authors:** Baojie Zhang, Dongyang He, Yu Fan, Nan Liu, Yijun Chen

**Affiliations:** State Key Laboratory of Natural Medicines and Laboratory of Chemical Biology, China Pharmaceutical University, Nanjing, China; University of Ulster, United Kingdom

## Abstract

Exenatide is an FDA-approved glucose-lowering peptide drug for the treatment of type 2 diabetes by subcutaneous injection. To address the issues on the inconvenience for patient use and the difficulty of oral administration of peptide drugs, chemical cross-linking of two pH-responsive biomaterials, alginate and hyaluronate, was carried out to prepare a new material for the encapsulation of exenatide as a form of microspheres. The exenatide-loaded microspheres exhibited spherical structures with excellent loading and release behaviors in the simulated gastrointestinal tract environments. After oral administration of the microspheres in *db/db* mice, maximum plasma concentration of exenatide appeared at 4 hours, and blood glucose was effectively reduced to a normal level within 2 hours and maintained for another 4 hours. The bioavailability of the exenatide-loaded microspheres, relative to subcutaneous injection of exenatide, reached 10.2%. Collectively, the present study demonstrated the feasibility of orally delivering exenatide with the new cross-linked biomaterial and formulation, and showed therapeutic potential for clinical applications.

## Introduction

Exendin-4 containing 39 amino acid residues was originally isolated from *Helodermatidae* venom. This peptide shares 53% sequence homology with glucagon-like peptide-1 (GLP-1) [1]. Given that exendin-4 can compete with GLP-1 for the same binding site in mammalian cells with higher affinity and stronger resistance to the degradation by dipeptidyl peptidase IV (DPP-IV), exendin-4 exhibits obvious advantages to be developed as a glucose-lowering agent with similar functions as GLP-1 [1]–[3]. Subsequently, exenatide, the synthetic version of exendin-4, was approved by the FDA in 2005 as adjunctive therapy to improve glucose homeostasis in type 2 diabetic patients [4]. However, the twice daily subcutaneous injections have posed clear shortcomings, such as inconvenience for the administration, local pain and irritation during the injection [5]. Therefore, it is favorable for exenatide to mimic physiological route of GLP-1 from intestine to circulation to avoid potential side effects [6]. Consequently, the development of a non-injective route for exenatide delivery has drawn great interests by both industry and academia, and oral administration is an ultimate choice based on the easy acceptance by diabetic patients [7].

Generally, protein and peptide drugs are rapidly denatured or degraded by low pH enviornment of the gastric media or the hydrolytic enzymes in the gastrointestinal tract [8]. In addition, the tight intestinal epithelium is a major barrier to block the absorption of macromolecular drugs [9]. As a result, protecting the integrity of protein and peptide drugs in stomach and promoting the absorption of the macromolecules by intestinal epithelium are the key challenges to deliver these drugs by oral administration.

Despite significant developments in oral delivery technologies, successful oral delivery of protein or peptide drugs remains very limited. To address various issues associated with oral administration of macromolecule drugs, different approaches have been attempted in recent years, including chemical modifications by substitution, acylation or PEGlation to alter their physiochemical properties, the addition of other novel functions and the use of more efficient delivery carriers [10]. Among these approaches, biotinylation of exendin-4 reported by Jin et al achieved 3.95% apparent bioavailability after oral administration [11]. Meanwhile, modification of exenatide with PEG (20 kDa – 40 kDa) resulted in prolonged plasma half-time to approximately 1 h *via* subcutaneous injection [12]. More recently, a pH-sensitive nanoparticle system composed of chitosan and poly-(γ-glutamic acid) was developed for oral delivery of exendin-4. However, the self-assembled nanoparticles were not stable in stomach. Although the subsequent filling of the exendin-4 loaded nanoparticles in enteric-coated capsules could deliver sufficient amount of exendin-4 into the circulation, it took 8 h for the drug to be effective on lowering glucose level in animals [13]. This suggested that the delayed effects of this delivery system are not suitable for clinical application. To overcome these disadvantages and become more clinical relevant, different from previous approaches, we therefore chose chemical cross-linking of alginate and hyaluronate aiming to prepare exenatide-encapsulated microspheres for more effective oral delivery of exenatide.

As biodegradable anion polysaccharides, both alginate and hyaluronate have been widely used to encapsulate various drugs for controlled release and targeted drug delivery [14], [15]. In addition to the naturally occurring and nontoxic properties, alginate and hyaluronate possess pH-responsive characteristics. Typically, they can keep compact in acidic solution while be swelled in alkaline condition. These two enviornments can mimic the situations of stomach and small intestine respectively. According to their special characteristics, any compounds loaded by alginate or hyaluronate could presumably be protected in stomach and be released in small intestine. Surprisingly, when alginate and hyaluronate were simply mixed together, they were unable to self-assembly organized because of the repulsion between these two materials. Therefore, cross-linking of alginate and hyaluronate by chemical method could generate a novel material that synergistically maintains respective pH-responsiveness and the capability of encapsulating exenatide. After preparation of exenatide-loaded microspheres by cross-linking of alginate and hyaluronate, *in vitro* releases in the conditions mimicking gastrointestinal pH environments, *in vivo* pharmacokinetics (PK) and glucose-lowering effects in *db/db* mice were performed and compared. The results indicated that the exenatide-loaded microspheres could effectively deliver the drug to lower plasma glucose level after oral administration.

## Materials and Methods

### 1. Ethics statement

All experimental animals were cared according to the guidelines of National Institute of Health (NIH), and animal studies were reviewed and approved by the Ethical Committee on Animal Experimentation of China Pharmaceutical University.

### 2. Materials

Sodium alginate (Mw: approx.180 kDa, pharmaceutical grade) was a gift from Huanghai Biological Pharmaceutical Co. Ltd (Qingdao). Sodium hyaluronate (Mw: 1,955 kDa, pharmaceutical grade) was purchased from Freda Biotechnology Co. Ltd (Shandong). Exenatide was from GL Biochem Ltd (Shanghai). Blood glucose meter and test strips were purchased from Johnson & Johnson Company (USA). Exendin-4 enzyme immunoassay kit (EK-070-94) was purchased from Phoenix Pharmaceuticals Inc. (USA). Bicinchoninic acid (BCA) protein assay kit was purchased from Generay Biotech Co. Ltd (Shanghai). All other chemicals were pharmaceutical grade and commercially available.

### 3. Animals

C57BL/ksJ *db/db* mice (male, 8–12 weeks) were purchased from Cavens Lab Animal Co. Ltd (Changzhou). According to the guidelines of NIH, animals were housed in the specific pathogen free (SPF) laboratory with clean sawdust. Mice were grouped 5 animals per cage and raised under 12 hours light or dark cycle, allowed water and food *ad libitum* which were replaced frequently to keep fresh. Cages and bottles were cleaned twice a week with changing new sawdust when necessary. After 2-week acclimatization, animal studies were performed and minimization of animal sufferings was undertaken during the experiments. Mice were gently grasped and quickly administered the drugs using smaller syringe or blood sample were extracted by thinner capllary tube. In the end of the experiments, mice were sacrificed between 5-8 weeks after the experiments by removal of cervical spine.

### 4. Preparation of exenatide-loaded microspheres

The dispersed phase consisting of 75 mg sodium alginate, 25 mg sodium hyaluronate and 3 mg exenatide was dissolved in 5 ml purified water and prepared by magnetic stirring at 400 rpm for 30 min. Then, it was supersonically vibrated for 20 min and held for 1 h to remove the bubbles. Afterward, 10 ml paraffin liquid, the continuous phase, was blended with 2.5% (v/v) Span-80 for 30 min at 200 rpm. Under magnetic agitating at 400 rpm, the dispersed phase was dropped into continuous phase at a speed of 2 drops per second from a needle tube to form droplets. After 30 min, 5 ml 1 N HCl and 5 ml 25% glutaraldehyde (GTA) were added to mediate the cross-linking of alginate and hyaluronate at 40°C and the formation of solid microspheres. Finally, the exenatide-loaded microspheres were washed with ethanol, harvested by filtration under reduced pressure and dried overnight at room temperature.

### 5. Characterization of the microspheres

The blank microspheres were characterized with scanning electronic microscope (SEM, S-3400N, Hitachi, Japan). Residual amount of GTA in the microspheres was analyzed by GC (column: HP-5; instrument: Agilent 6890 Series GC system), in which both GTA sample (0.01%) and the microsphere sample (10 mg/ml) were measured with a temperature gradient. Briefly, initial temperature was 100°C and maintained for 1 min, then rose to 180°C at the speed of 10°C per min and held for 30 min. Finally, the column was kept at 280°C for 5 min. Temperatures of sample injector and detectors were 210°C and 250°C respectively.

The drug loading efficiency (LE) and loading content (LC) of the microspheres were determined by the amount of free exenatide remained in the supernatant using BCA protein assay kit [16] and calculated using the following equations [13]: 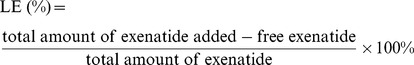


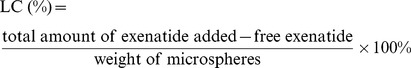



### 6. Accumulative releases of exenatide

To simulate the environments of stomach and small intestine, 100 mg exenatide-loaded microspheres were distributed in 10 ml of 0.063 N HCl solutions (pH 1.2) for 2 h followed by transferring to 10 ml of 0.1 M sodium phosphate solution (pH 7.4) at 37°C. A solution of 500 µl was exactly pipeted from the upper layer every 1 h and filtered. At the same time, 500 µl of fresh warmed sodium phosphate buffer was added into the original solution. Exenatide in the supernatant was detected by BCA kit.

Swelling ratios (SR) at different time periods were investigated. Free water remained on the surface of swelled microspheres was dried by filtration under reduced pressure and SR was calculated as following [17]: 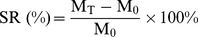



where M_T_ is the weight of swelled microspheres and M_0_ is the weight of initial dry microspheres.

### 7. Pharmacodynamics (PD) and PK study

All male *db/db* mice were fasted for 12 h and remained fasting during the experiments, but were allowed water *ad libitum*. Following experimental groups were designed and divided: blank microspheres *via* oral administration as a blank group; blank microspheres blended with free exenatide solution (500 µg/kg) *via* oral administration as a negative control group; exenatide-loaded microspheres (500 µg/kg) *via* oral administration as an experimental group; and free exenatide solution (50 µg/kg) *via* subcutaneous injection (SC) as a positive control group. To blend blank microspheres and free exenatide, exenatide was firstly dissolved in physiological saline, and blank microspheres were added to this solution to form a mixture. Each group contained 8 *db/db* mice. Blood samples were collected from the tail veins of mice prior to the experiments and every 1 h or 2 h during the study, and blood glucose concentrations were measured by glucose meter.

To analyze the concentration of exenatide in plasma at different time points, blood samples were drawn from orbital sinus of mice and centrifuged (5000 rpm, 4°C for 45 min) prior to experiments and at predeterminate intervals of 1 or 2 h during the experiments. The plasma concentration of exenatide was quantified with exendin-4 ELISA kit. The relative bioavailability (BA_R_) of the microspheres was calculated by the following formula [13]: 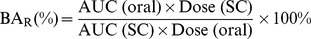



where AUC represents the area under the curve of plasma exenatide concentration versus time.

### 8. Statistical analysis

Data were shown as mean ± standard deviation (SD). Comparison between two groups was analyzed by two-tail t-test with the statistical software (SPSS). A significant difference was defined as P < 0.05.

## Results and Discussions

### 1. Preparation of exenatide-loaded microspheres

Microspheres were produced by initial emulsion and then suspended in GTA solution for cross-linking [18]. After dissolution of alginate, hyaluronate and exenatide in the dispersed phase, the resulting mixture was dropped into the continuous phase to form microspheres. As shown in Table 1 and Table 2, mass ratio of alginate and hyaluronate to be 3:1 and concentration of the dispersed phase to be 20 mg/ ml resulted in the best productivity of the microspheres and loading efficiency. According to previous report, if the dispersed phase dissolved evenly in the continuous phase, the formed microspheres would have a uniform appearance. Thus, the proportion between the dispersed and continuous phase was a key factor [19]. In our case, the examination of the ratio of dispersed phase to continuous phase in three different groups resulted in similar productivity and loading efficiency. Therefore, we chose the ratio of 5:10 for subsequent experiments to minimize the use of paraffin liquid. Since surfactant was able to reduce the surface tension of continuous phase, an appropriate surfactant was chosen based on the hydrophilicity-lipophilicity balance (HLB). Given the fact that the type and amount of surfactant could significantly affect the drug loading and release behaviors of the microspheres [20], 2.5% Span-80 was identified to achieve the best loading efficiency (Table 3 and Table 4).

**Table 1:** Comparison of different dispersed phase^a^.

	Dispersed phase (mg/ml)		
	40	20	10
Productivity (%)	63.3±0.85	68.7±1.71	64.3±0.98
LE (%)	58.3±2.98	72.9±1.57	69.8±0.84

a Data are averages of three independent experiments with standard deviations.

**Table 2:** Comparison of different mass ratio between alginate and hyaluronate^a^.

	Alginate:Hyaluronate (w/w)			
	1∶1	1∶2	2∶1	3∶1
Productivity (%)	58.2±1.91	50.1±1.19	61.4±0.74	67.9±1.33
LE (%)	60.7±0.74	55.4±1.54	64.5±2.64	70.9±1.71

a Data are averages of three independent experiments with standard deviations.

**Table 3:** Comparison of volume ratio between dispersed phase and continuous phase^a^.

	Dispersed phase: Continuous phase (v/v)		
	5∶50	5∶25	5∶10
Productivity (%)	63.8±2.12	64.7±0.69	64.2±1.17
LE (%)	72.1±0.59	70.4±1.75	71.6±0.87

a Data are averages of three independent experiments with standard deviations.

**Table 4:** Comparision of different surfactant concentration^a^.

	Surfactant Concentration (%)			
	0.5	1.0	2.5	5.0
Productivity (%)	65.4±1.62	65.5±2.94	66.7±0.57	61.7±2.45
LE (%)	60.3±2.94	66.7±2.41	72.1±1.65	71.7±0.49

a Data are averages of three independent experiments with standard deviations.

To cross-link alginate and hyaluronate, GTA, a widely used and effective cross-linking agent was used to form a hemiacetal between alginate and hyaluronate in the presence of HCl [21]. Because the degree of cross-linking increased with longer exposure time to GTA [22], we prepared three different types of microspheres by changing GTA reaction time as 0.5 h, 1 h and 2 h. Subsequently, comparison of different reaction time resulted in significantly different release behaviors due to the changes in size and appearance of the microspheres. As shown in Table 5, the microspheres prepared by using 1N HCl and 5 ml GTA at 40°C exhibited extended release of exenatide and gave the best accumulative release of exenatide. Taken together, optimization of pH value, GTA amount, reaction temperature and reaction time produced satisfactory microspheres suitable for the encapsulation and release of exenatide.

**Table 5:** Release behaviors of exenatide-load microspheres under optimized cross-linking conditions^a^.

Time (h)	1N HCl	5 ml GTA	40°C
1	4.43±1.41%	5.67±1.42%	4.67±1.89%
2	11.63±2.83%	9.87±1.99%	10.5±3.53%
3	43.60±2.76%	40.53±2.12%	45.57±1.07%
4	64.60±1.07%	67.23±1.91%	66.37±2.84%
5	80.70±1.07%	85.53±0.79%	86.37±2.32%
6	92.43±1.41%	96.73±2.35%	96.47±1.77%
7	100.0±2.49%	100.0±3.29%	100.0±2.97%

a Data represent the percentage of released amount of exenatide from the microspheres by cross-linking for 0.5 h and are averages of three independent experiments with standard deviations.

### 2. Characterization of the microspheres

**Figure 1:**
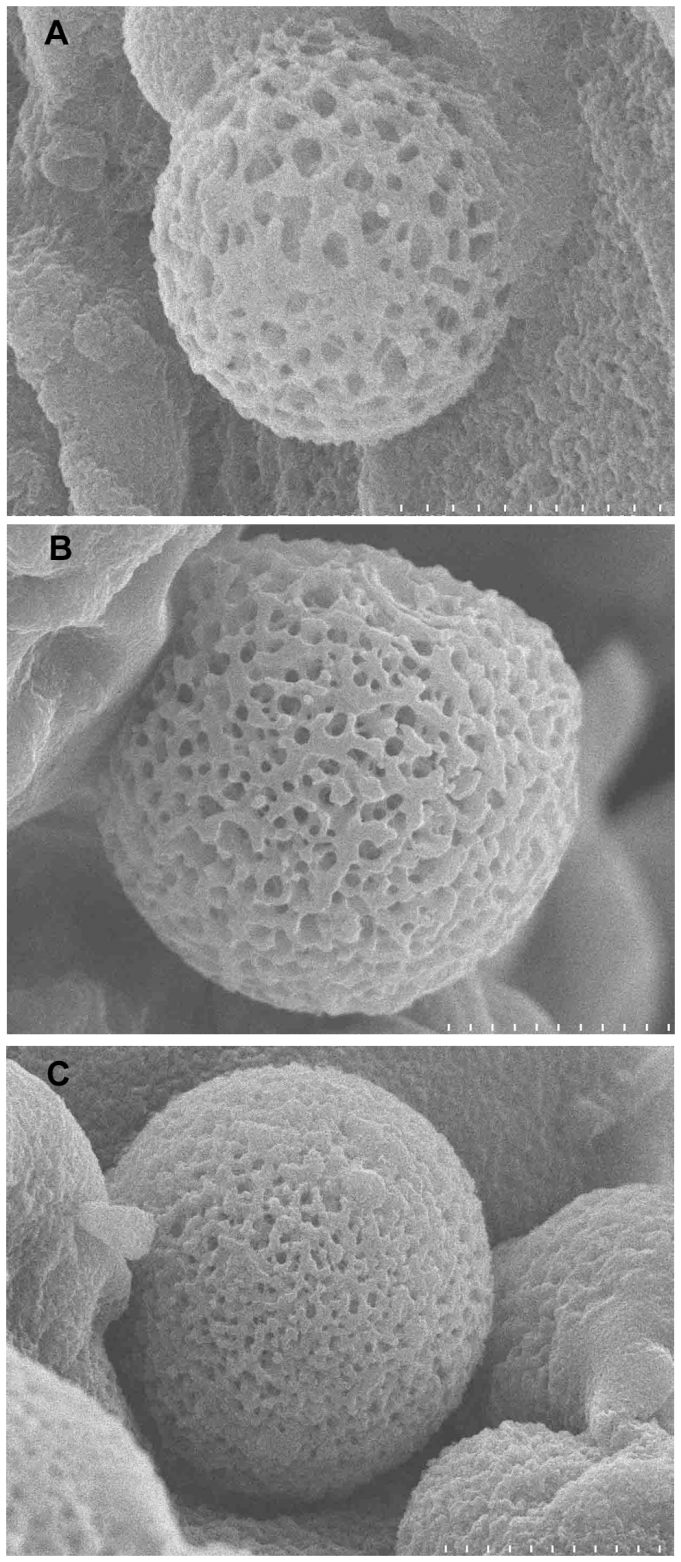
SEM micrographs of microspheres cross-linked with alginate and hyaluronate. (A) Micrograph of cross-linking for 0.5 h with a measurement unit of 4 µm fewer than 12,000 magnifications. (B) Micrograph of cross-linking for 1 h with a measurement unit of 10 µm fewer than 4,000 magnifications. (C) Micrograph of cross-linking for 2 h with a measurement unit of 10 µm fewer than 4,000 magnifications.

The loading efficiency for exenatide in the microspheres was 71.4±2.57% (n = 3) and loading content was 2.98±0.91% (n = 3). Same as the microspheres prepared by other materials, the present microspheres prepared with cross-linked alginate and hyaluronate also showed a globular structure (Fig. 1.). However, they exhibited porous holes on the surface, which was significantly different from the microspheres prepared with alginate or hyaluronate alone [18], [23]. The average diameter of the microspheres cross-linked for 0.5 h was approximately 4.1 µm, whereas the microspheres cross-linked for 2 h reached 15 µm. At the same time, the holes on the surface of the microspheres cross-linked for 2 h were much smaller than those cross-linked for 0.5 h and 1 h. Consequently, the time taken to release the drug from the microspheres cross-linked for 2 h was significantly longer than that for 0.5 h.

**Figure 2:**
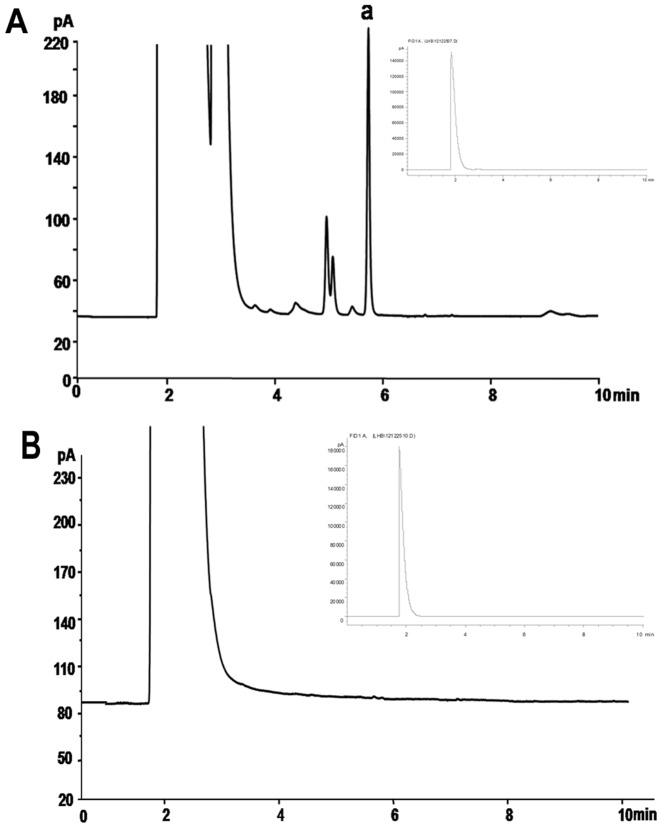
GC analysis of residual GTA content in the microspheres. (A) Partially enlarged GC chromatogram of 0.01% GTA. Top right corner shows the full chromatogram. (B) Partially enlarged GC chromatogram of blank microspheres (10 mg/ml). Top right corner shows the full chromatogram.

Because GTA has the potential to produce the cross-links between DNA and proteins, it could increase the frequency of airway symptoms in humans [24], [25]. Therefore, residual GTA amount in the microspheres was determined by gas chromatography (GC). No residual GTA in the blank microspheres was observed from GC analysis (Fig. 2), suggesting that residual GTA was not an issue in the present microsphere preparation.

### 3. Accumulative releases of exenatide

**Figure 3:**
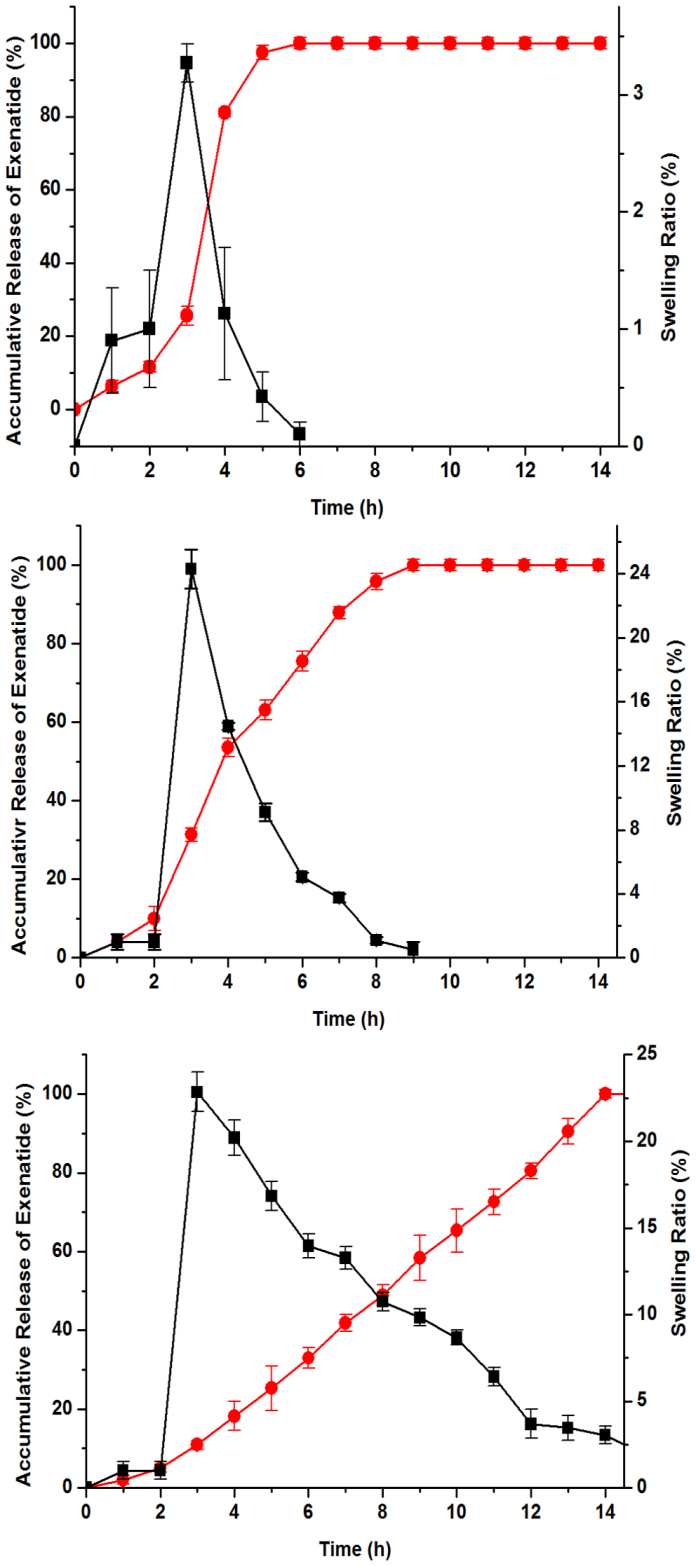
Dissolving and swelling characteristics of different microsphere preparations. (A) Cross-linking for 0.5 h. (B) Cross-linking for 1 h. (C) Cross-linking for 2 h. All black squared curves depicted by line and symbol represent the changes of swelling ratio and red circled curves show the changes of exenatide release. The experiments were conducted in 0.063 N HCl solutions (pH 1.2) for the first 2 h and then transferred to 0.1 M sodium phosphate solution (pH 7.4). Data are averages of three independent experiments and error bars represent standard deviations.

The swelling pressure inside polymeric network can be enhanced due to electrostatic repulsion between charged moieties. Additionally, charge mobility can also be hindered by covalently cross-linked polymer [26]. As a result, higher osmotic pressure at internal gel and lower osmotic pressure at external solution could lead to the trend from solvent into the gel, and the cross-linked structure could be stable during the process of swelling [27]. Once the swelling degree reached the highest level, the microspheres would start to lose weight and the polymeric structure would begin to disintegrate [28]. In consistent with such a process, a close correlation between swelling ratio of the microspheres and released exenatide could be observed. In the group of microspheres cross-linked for 0.5 h, the swelling ratio was around 3.5 after 1 h switching from HCl solution (pH 1.2) to sodium phosphate saline (pH 7.4), and the accumulated exenatide concentration reached 80%. After another hour, complete release of exenatide was achieved at pH 7.4 (Fig. 3A). In the case of cross-linking for 1 h, swelling ratio reached approximately 15.3 in sodium phosphate saline (pH 7.4), and 31.3% exenatide was released at this time period (Fig. 3B). Very differently, the microspheres by cross-linking for 2 h reached a swelling ratio of nearly 25 after 1 h switching from pH 1.2 to pH 7.4. Meanwhile, exenatide was only released less than 20%, and it took 12 h for the complete release (Fig. 3C). This comparison of cross-linking time indicated that cross-linking for 0.5 h exhibited the best swelling ratio and release profile.

### 4. In vivo PD and PK

**Figure 4:**
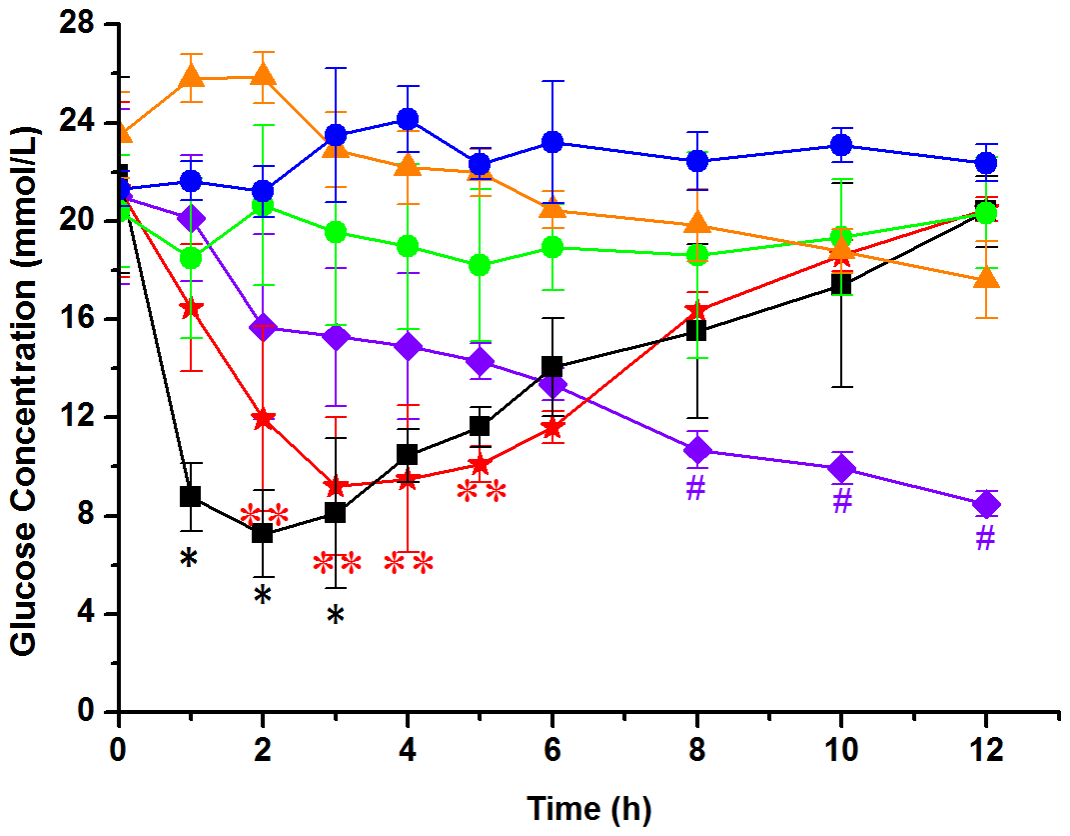
Glucose-lowering effects by exenatide-loaded microspheres in *db/db* mice. Blank microspheres (blue circle); Blank microspheres blending with free exenatide solution *via* oral delivery (500 µg/kg) (green circle); Subcutaneous injection of free exenatide solution (50 µg/kg) (black square); Oral administration of exenatide-loaded microspheres (cross-linking for 0.5 h, 500 µg/kg) (red star); Oral administration of exenatide-loaded microspheres ( cross-linking for 1 h, 500 µg/kg) (violet rhombus); Oral administration of exenatide-loaded microspheres (cross-linking for 2 h, 500 µg/kg) (orange triangle). Statistical difference between treated group and untreated group (green circle) was indicated as black asterisk, double red asterisk and violet hash under the error bars (P<0.05). Each group consisted of 8 *db/db* mice.

As shown in Fig. 4, the group treated with subcutaneous injection of exenatide effectively lowered blood glucose within 1 h and kept effective for another 2 h, and then gradually returned to the original level. The glucose concentration in the blank microsphere group kept greater than 20 mmol/L after oral treatment. Similialy, there was no obvious effect to lower glucose level in the blank microspheres blended with free exenatide, indicating that free exenatide was completely destroyed in stomach. In contrary, the groups orally treated with exenatide-loaded microspheres exhibited remarkable glucose lowering effects, but different groups showed significant difference. The group of cross-linking for 0.5 h started to be effective at 2 h after oral administration and maintained the effectiveness for another 4 h, whereas the group of cross-linking for 1 h did not lower blood glucose until 8 h after oral administration. For the group of cross-linking for 2 h, the effect on glucose lowering was not observed, suggesting that the release speed of exenatide from highly cross-linked microspheres was very slow or the concentration of exenatide absorbed by intestine was too low to be effective. These results further confirmed that microspheres prepared by cross-linking of alginate and hyaluronate with a specific cross-linking degree could be a valuable vehicle for the oral delivery of exenatide. Moreover, compared to previous microspheres prepared by loading palmityl-acylated exendin-4 in porous poly-lactic-co-glycolic acid for long-acting hypoglycemic effect [29], the present microsphere system does not have the issue on the requirement to tolerate inhaling therapy by the patients.

**Figure 5:**
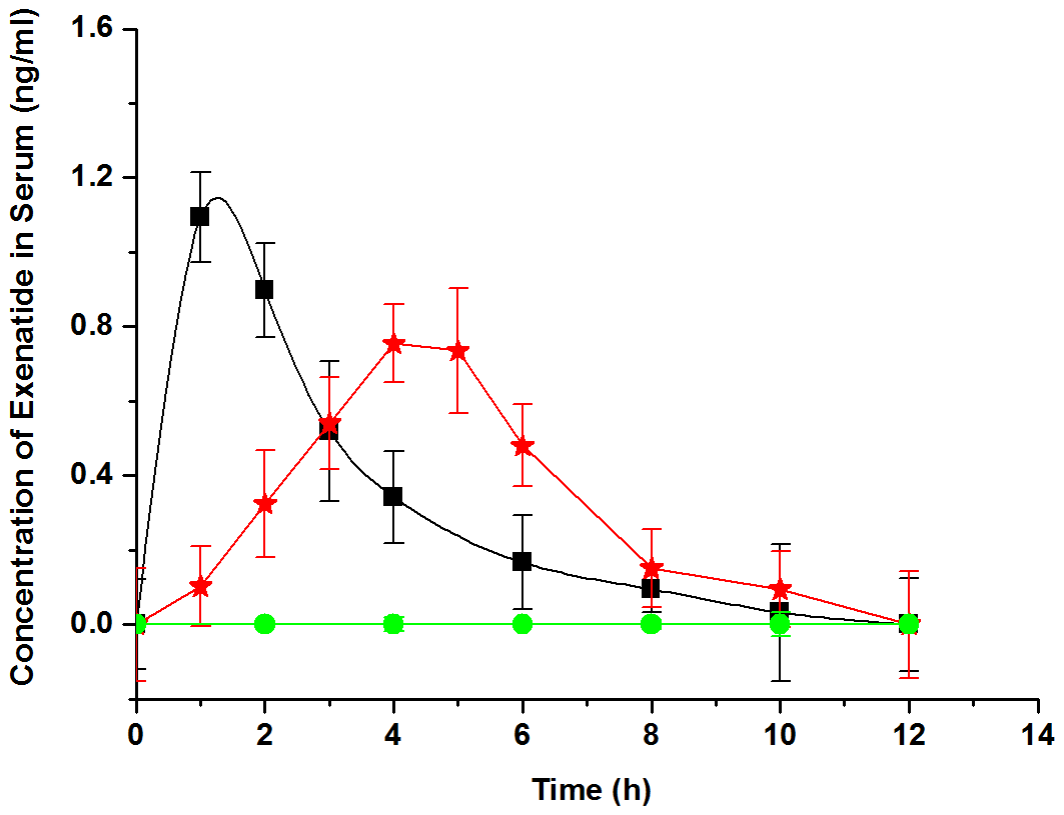
Pharmacokinetic study of exenatide-loaded microspheres in *db/db* mice. Free exenatide solution administered by subcutaneous injection (50 µg/kg) (black square); Oral administration of blank microspheres blending with free exenatide solution (500 µg/kg) (green circle); Oral administration of exenatide-loaded microspheres (cross-linking for 0.5 h) (red star). Each group consisted of 8 *db/db* mice.

To determine the pharmacokinetic parameters, the plasma exenatide concentration was measured after orally administrating exenatide-loaded microspheres. As shown in Fig. 5, there was no detectable exenatide in the group of orally administrating blank microspheres blended with free exenatide, whereas the plasma exenatide concentration reached its maximum in the subcutaneously treated group at 1 h post-injection with AUC value of 3614±183 pg h/ml. Even though the maximum plasma exenatide concentration of the group orally treated with exenatide-loaded microspheres by cross-linking for 0.5 h appeared at 4 h after the treatment, this group behaved similarly to subcutaneous injection in terms of AUC value (3667±19 pg h/ml). The relative bioavailability of oral administration of this exenatide-loaded microsphere system was calculated to be 10.15±3.51%, which is close to the BA_R_ reported by Nguyun et al [13]. Because of the relatively low bioavailability by oral delivery, a 10-fold increase of doing was required to achieve similar effects. Nevertheless, these PK parameters correlated well with the glucose-lowering effects mentioned above. The present PD/PK profiles demonstrated that the exenatide-loaded microspheres were stable and intact in the acidic environments of stomach, thereby promoting exenatide arriving at small intestine. Once entering small intestine, the microspheres begin to swell and release loaded exenatide. Moreover, it was noted that the size and shape of the microspheres are critical to the release and successful delivery of exenatide by oral route.

In the present study, the exenatide-loaded microspheres cross-linked for 0.5 h could effectively mimic the effects of subcutaneous injection of exenatide. Compared to reported nanoparticles and microspheres for oral delivery of exenatide [11], [29], the present oral delivery system showed comparable effectiveness as subcutaneous injection, more desirable PK profile and convenient preparation and administration. Although the exenatide-loaded microspheres showed certain advantages for potential clinical application in the present study, the safety of the microspheres for human use requires further systemic evaluation.

## Conclusion

After cross-linking of alginate and hyaluronate in the presence of exenatide, the exenatide-loaded microspheres were successfully developed as an oral delivery vehicle for exenatide. Through pH-responsive mechanism, this novel vehicle could keep intact in stomach and then release loaded exenatide in small intestine for absorption. The PD/PK profiles of the present oral vehicle exhibited the comparable effects on lowering blood glucose and kinetic behaviors as subcutaneous injection. Thus, the present study on the microspheres has the potential to substitute subcutaneous injection and provides an alternative therapeutic tool for diabetic patients.
